# Supplementary material for: Are carbon emissions trading and green financial instruments synergistic? -Comprehensive quantitative research based on content analysis
Source: PLoS One. 2024 Mar 7;19(3):e0298601. doi: 10.1371/journal.pone.0298601 (PMC10919601; doi:10.1371/journal.pone.0298601)
Supplement: S1 Data — (ZIP) [file pone.0298601.s001.zip › Data/地级市Z_F工作报告.zip!/║■─╧/╙└╓▌╩╨/└┤╫╘╣1⁄2╓┌║┼í╛┬φ┐╦╩2╛▌═°í┐_2017.docx]

各位代表：

现在，我代表市人民政府，向大会报告政府工作，请予审议，并请各位政协委员提出意见。

一、过去五年工作回顾

过去五年，全市人民在市委的坚强领导下，坚持以全面建成小康社会为总揽，扎实推进品质活力永州建设，较好完成了市四届人大一次会议确定的各项目标任务，全市经济社会发生了深度而有品质的变化。

经济发展迈入跨越赶超新阶段。主要指标稳步提升。2012年至2016年，全市地区生产总值由945．4亿元增加到1540．5亿元（预计数，下同），年均增长9．4％；规模工业增加值由261．8亿元增加到340亿元，年均增长10．8％；固定资产投资由593．3亿元增加到1645．9亿元，年均增长24．2％；社会消费品零售总额由285．2亿元增加到589．6亿元，年均增长13．2％；财政总收入由70．5亿元增加到150亿元，年均增长16．3％，总量由全省第10位上升到第8位。金融机构各项存款余额由845亿元增加到1900亿元、贷款余额由401．6亿元增加到970亿元，分别增长124％和141％。产业培育成效明显。规模工业企业由666家发展到865家，产值过亿元企业由92家发展到400家，长丰汽车、零陵烟厂建成年产能超50亿元企业，装备制造、农产品加工、矿产品加工、轻纺制鞋、电子信息建成百亿产业，永州经开区、祁阳经开区、江华经开区、宁远工业园、冷水滩高科园建成百亿园区；农业产业化走在全国先进行列，成功跻身全国油茶产业发展示范市，一大批县区被列为全国粮食、蔬菜、生猪生产重点县区，省级以上农业产业化龙头企业发展到46家，农产品加工业销售收入突破500亿元；文化旅游、电子商务、商贸物流、金融保险、房地产等第三产业发展提速，全市等级旅游景区达到22家，市级金融保险服务机构发展到67家，“互联网＋”领域新业态成为经济发展新的增长点，三次产业结构由24．1：38．3：37．6调整为21：35：44。创新驱动能力增强。先后实施省级以上科技项目297项，高新技术企业由64家发展到241家，高新技术产业产值占规模工业总产值比重达到25％，新增一批“国字号”质量、商标品牌及省级质量奖项，成功入选国家知识产权试点城市。县域经济加速崛起。地区生产总值过百亿县区达到7个、过两百亿县区3个，财政总收入过10亿县区6个、过15亿或接近15亿县区3个。江华、宁远等县区经济总量在全省排位前移，冷水滩进入全省首批全面小康达标县区。

城乡建设迈上提质升级新台阶。中心城区“三年扩容提质行动”带来巨变。建成区面积由54平方公里拓展到62．2平方公里，人口由52．8万增加到63．6万，基本形成中等城市框架。成功创建国家卫生城市、国家森林城市，国家历史文化名城获国务院批准。县城和小城镇建设全面提速。纷纷变大、变美、变新、变绿、变畅，辐射带动能力显著增强。江华再获全国文明县城称号，宁远、东安、新田成功创建国家卫生县城，祁阳县、东安县芦洪市镇获批国家新型城镇化综合试点地区，回龙圩等13个乡镇入选全国重点镇。全市城镇化率由37．9％提高到47％。美丽乡村建设让农村生机勃发。全面推进农村环境综合治理，先后整治重点村1538个，创建国家、省级美丽乡村28个，5个村入选国家历史文化名村，　24个村入选中国传统村落。全市农村卫生清扫保洁率达到86％，生活垃圾基本实现集中收运。重大项目推动基础设施强力突破。二广高速、厦蓉高速出省通道全面打通，祁冷一级公路和207国道改建双牌至道县段竣工通车。10个县区开通高速公路，所有行政村通水泥（沥青）路。经停永州高铁达到17对，永州机场新增上海、海口航线。涔天河水库扩建工程下闸蓄水、灌区工程全面推进，毛俊水库开工建设，1098座病险水库除险加固全面完成。神华国华永州火电进展顺利，新增水电装机21万千瓦，风电开发规模全省第一，城乡电网不断完善。“智慧永州”建设加速推进，成功入选国家“电信普遍服务试点城市”，祁阳获批国家“智慧城市”试点县。

改革开放迈开创先领先新步伐。行政管理体制改革不断深化，“三清单一目录”管理制度基本确立，市本级先后取消行政许可项目15项，下放县区行政许可事项130项，非行政许可审批彻底终结；市、县区政府机构改革全面到位，蓝山获批省直管试点县；在全省率先实施“先照后证”“三证合一”改革，极大地激发了市场主体活力。农村产权制度改革深入推进，宁远县发放了全省第一本农村土地经营权证，新田县入围全国农村承包土地经营权抵押贷款试点县。社会治理机制改革落地见效，城乡网格化治理全面推进，“步行半小时服务圈”基本建成。财税金融与投融资体制改革取得突破，农村信用社改制组建农村商业银行全面完成，市县区投融资公司融资能力显著提升。国有林场改革、并乡合村改革、供销合作社综合改革走在全省前列。国企改革全面到位。全力推进承接产业转移示范区建设，开放开发活力迸发。五年累计承接产业转移项目1857个，引进内资902亿元、外资36．8亿美元，完成外贸进出口总额35．4亿美元。新增3个省级出口基地，7个县区获批国家出口食品农产品质量安全示范区。海关、商检永州办事处相继设立，公路口岸、铁路口岸获批建设，湖南（道县）农副产品集中验放场试运行。

民生事业迈进全面小康新征程。全面小康实现程度由77．6％提高到87．9％，五年提高10．3个百分点，多次荣获全省全面小康“管理成效奖”。城乡居民收入分别达到24022元、11734元，分别是2011年的1．4倍、1．96倍。社会保障水平稳步提高。就业优先成效显著，五年新增城镇就业29．7万人、农村劳动力转移就业29．5万人，帮助下岗失业人员再就业12．2万人，发放创业小额担保贷款16．7亿元。各项社会保障制度实现城乡全覆盖，五项保险参保达到614．2万人次，支付待遇232．52亿元。投入资金308．6亿元，解决了12．54万户住房困难家庭的居住问题；提取及发放公积金贷款96．8亿元，支持缴存职工购建住房面积630万平方米。投入资金9．35亿元，解决了193．4万人的饮水安全问题；投入资金18．24亿元，解决了60．8万人的脱贫问题。各项社会事业协调发展。教育优先得到确立，投入资金64亿元，新增城区学位8．51万个，564所农村完小以上学校全部建成合格学校，“三名工程”成效显著，各类教育协调发展，高考成绩屡创新高。零陵区获评国家义务教育均衡发展县区。生态优先日益凸显，湘江源头区域获批国家生态文明先行示范区，5个县区列入国家主体功能区建设试点示范，7个县区列入国家重点生态功能区，新增2个国家级自然保护区、1个国家森林公园、8个国家湿地公园、3个国家水利风景区，湘江保护和治理第一个“三年行动计划”圆满完成，节能减排目标全面实现。文化强市扎实推进，文体惠民工程深入实施，文化遗产保护全面加强，新增全国重点文物保护单位19处、国家级非物质文化遗产项目4个，柳子街入选全国首批历史文化街区，成功举办两次全省和一次全市公祭舜帝大典。精神文明建设成效显著，“欢乐潇湘·和美永州”等文体活动精彩纷呈，奥运冠军、残奥会冠军实现零的突破。医疗卫生事业快速发展，投入资金11．5亿元，新建改建扩建各级各类卫生计生机构1265处，市妇幼保健院顺利搬迁，市中心医院、市中医院成功创建“三甲”医院，主要卫生资源指标实现翻番，中医药事业加速发展。计生工作连年“保先进位”，　3个县区获评全国计划生育优质服务先进单位。社会治安大局持续稳定。依法治访全面推进，信访秩序不断改善，进京非访登记人数总量退出全省前五。群体性事件、复杂矛盾纠纷和各类安全隐患稳妥处置，严重暴力刑事案件逐年大幅下降，打击毒品犯罪、食药犯罪、网上追逃等重大专项行动成果全省领先，现行命案侦破和命案积案侦破分获全省“四连冠”“五连冠”，公众安全感和政法队伍满意度稳居全省前列。食品药品安全稳中向好。安全生产五年获评全省先进。

民主法制迈向依法治市新时期。始终坚持依法行政，扎实推进法治政府建设，健全完善各项规章制度，有序开展立法工作，依法履职能力不断增强。认真执行市人大及其常委会的决议、决定，自觉接受市人大及其常委会的法律监督、工作监督，主动接受人民政协的民主监督和社会舆论监督。五年办理人大代表议案、建议974件，政协委员提案1062件，解决了一大批人民群众关心关注的实际问题。大力推进简政放权、放管结合、优化服务，创新政务公开方式，加快电子政务建设，“政务云”初具规模，政务服务效能大幅提升。切实加强基层民主，有序推进普法教育，“六五”普法全面完成。不断强化行政监察和审计监督，健全完善政府绩效考评，切实加强反腐倡廉，积极推进公车改革，政风行风不断好转，政府执行力和公信力不断增强。统筹做好民族宗教、外事侨务、新闻出版、统计、移民、科协、气象、地震、档案、对台、老龄、人防、关工委、残联、慈善、红十字会等工作，工会、共青团、妇联等群团组织作用充分发挥。大力支持国防和军队改革，驻永部队和民兵在全市经济社会发展中作出积极贡献，获评全省双拥模范城。

2016年，是本届政府履职最后一年。我们认真贯彻落实五大发展理念和市委决策部署，全市经济社会发展取得新成效，实现了小康决胜和“十三五”发展开门红。

精准发力稳增长，经济发展稳中向好。制定实施推进供给侧结构性改革促进经济持续稳定增长的系列政策措施，加大企业帮扶力度，科学调度经济运行，保持了经济平稳向好，主要指标增速多数保持全省第一方阵。预计，地区生产总值增长8％，规模工业增加值增长6．4％，固定资产投资增长13．8％，社会消费品零售总额增长11．7％，财政总收入增长14．3％，城乡居民可支配收入分别增长9．5％、9％。全面小康实现程度提升3．3个百分点。外贸进出口总额完成9亿美元，实际利用外资9．35亿美元，内联引资220亿元。非公有制经济平稳健康发展。

坚定不移促转型，产业升级成效初显。新型工业在创新驱动中提质增效。完成工业固定资产投资737亿元、技改投资316亿元。新增高新技术企业21家，永州经开区跻身省级高新技术开发区。长丰猎豹生产整车6万台，完成产值50．2亿元，分别增长58％、80％。新入统规模工业企业130家，居全省前列。工业税收增幅达到20．5％，占全市税收比重由33％提高到35％。军民融合步伐加快，一批优秀民企相继融入军工领域。“135工程”新建标准厂房166万平方米，新入园企业193家。园区规模工业总产值增长20．4％。现代农业在委市共建中步伐加快。与省农业委合作共同推进农业标准化建设，新增农业标准化示范基地62个，省级农业产业化龙头企业11家、现代农业特色产业示范园16家，省著名商标28件，“三品一标”产品118个。实现粮食总产340万吨，收购烟叶60．55万担，养殖业总产值突破350亿元。永州国家农科园通过科技部验收，向全国先进行列迈进。服务业在消费升级中持续繁荣。全域旅游来势较好，全市接待游客4239．9万人次，实现旅游综合收入228．3亿元，分别增长17．9％和19．8％。东安、江永、宁远跻身全国全域旅游示范区创建单位，金洞进入国家森林康养基地试点，舜皇山成功创建国家4A级景区。房地产业持续健康发展，全市房屋交易面积和成交额分别增长4．5％和52％。电子商务发展势头强劲，新入统省电子商务系统企业240余家，从事电商业态人员达10余万人，全年电商交易额突破140亿元。

持之以恒创品牌，城乡面貌持续改观。中心城区建设再展新颜。全年开工城建项目130个，完成投资100亿元。零陵古城、“两中心”、湘南万商红商贸新城、永州大道地下综合管廊及后续配套等项目开工建设，滨江新城路网及安置小区、湘江西岸棚户区改造、宋家洲综合整治、BRT快速公交等项目加快推进；湘江东路、双洲路、翠竹路、零陵路、人民路等建筑立面改造，永州大道城区段、潇湘大道、长丰大道、南津路、潇水东路等主干道提质改造基本完成，源味街开街运营。创建全国文明城市、国家交通管理模范城市、国家园林城市工作有序推进，获批省地下综合管廊建设试点城市。县城、小城镇和美丽乡村建设齐头并进。完成县域城镇建设投资80多亿元，县城扩容提质取得新成效，面貌发生新改观；品质活力示范乡镇建设稳步推进，省级美丽乡镇示范镇创建实现零突破。1550个村庄规划编制、64个美丽乡村、512个环境整治重点村建设整治任务全面完成。基础设施建设持续发力。冷东一级、零双一级公路开工建设，新增二级公路53公里，改造农村公路1100公里。开工建设农林水重点项目41个，实施并完成小农水项目县6个、病险水库改造552座、“五小”水利工程7190处。完成电网建设改造投资6．4亿元。神华国华永州火电完成投资21亿元。华为云计算中心一期业务正式运营，电信普遍服务试点项目顺利推进，新增700个行政村通宽带网络，中心城区公共场所实现无线网络全覆盖。生态永州建设成效明显。国家生态文明建设示范市创建工作启动。完成造林面积35．21万亩，森林覆盖率提高到64．5％。提前一年完成“美丽湿地”三年行动计划。湘江保护与治理第二个“三年行动计划”顺利实施。小一型以上水库污染治理全面完成。3个县获评全国国土资源节约集约模范县。

蹄疾步稳抓改革，制度红利加速释放。供给侧结构性改革扎实推进。淘汰冶炼企业22家、关闭停产143家；消化商品房库存483万平方米，盘活土地库存1．7万亩；到位政府债务置换资金96亿元、政策性银行贷款62亿元，非法集资防控处置力度加大；阶段性降低了企业职工养老、失业、工伤保险等社保缴费比例，减免行政事业性收费1．7亿多元，“营改增”为企业减税5亿多元。企业“五证合一、一照一码”和个体工商户“两证整合”登记制度全面实行，新设立市场主体3．43万户、注册资本413．3亿元。行政管理体制改革大力推进。市本级新取消和下放行政事务管理职责17项。整合建立全市统一的公共资源交易平台基本完成。市、县两级不动产登记中心成立，实现全域发证。市政务服务中心获批国家社会管理和公共服务标准化试点。乡镇区划调整改革圆满完成，全市共撤并乡镇35个，撤并建制村2100个。农村产权制度改革深入推进。县区农地利用规划编制基本完成，农村承包土地经营权抵押贷款稳步推进，县、乡土地流转服务体系基本建立，新增土地流转面积83．7万亩。社会治理机制改革全面推进。推动15个部门69项公共服务事项下放到社区，44项公共服务事项进乡镇、43项进村，集社会治理、城市管理、公共服务于一体的信息平台上线运行，获评全国创新社会治理优秀城市。投融资体制改革积极推进。永州农商行挂牌营业，县区农建投、旅建投、园建投纷纷设立。全市新增银行贷款165亿元，资本市场直接筹资80亿元，市属三大平台公司到位融资130亿元。推出18个总投资468亿元的PPP项目吸引社会投资。奔腾文化创意公司获准在新三板上市。

共建共享惠民生，群众福祉不断增强。民生资金投入更多。民生支出占财政总支出比重达到79．8％，增长16．1％。省定16件重点民生实事和市定6件惠民实事全面完成。社会保障水平更高。新增城镇就业6．22万人、农村劳动力转移就业5．05万人、创业主体2．45万户，发放创业小额担保贷款3．6亿元。企业退休人员养老金月人均水平达到1890元，城乡居民基础养老金最低标准调整到80元，医疗保险实现并轨。农村特困人员供养水平提高到每年3200元，城乡低保标准提高到每月420和220元。实施棚户区改造3．3万套、农村危房改造2．28万户。提取及发放公积金贷款31．5亿元，支持8000户职工解决住房问题。新增农村饮水安全受益人口30万。社会事业发展更快。新增中心城区义务教育学位8000个，新建义务教育合格学校109所、农村公办幼儿园27所，乡村教师支持计划全面实施，“三名工程”持续见效，高考一本、二本上线人数均创历史新高。潇湘技师学院搬迁新址办学，永州幼儿师范高等专科学校开工建设。城市公立医院和县级公立医院改革稳步推进，公立医疗机构药品零差率销售实现全覆盖，分级诊疗启动实施，全面两孩、孕产妇产前筛查城乡免费服务政策有效落实。公共文化体育服务体系进一步完善，公共文体场馆全面免费开放。《永州市志》、《舜帝陵志》和县区年鉴编修工作进展顺利。人防工作荣获“全国人民防空先进集体”。精准扶贫力度更大。全年减少贫困人口14．26万人，贫困村退出132个，贫困发生率下降4．5个百分点。“四跟四走”产业扶贫模式在全国推介。扶贫小额贷款发放超20个亿，全省第一。开工建设易地扶贫搬迁项目80个，完成搬迁3．96万人。建成贫困村电商村级服务站184个、金融扶贫服务站404个，实施村级光伏扶贫项目101个，九嶷山、勾蓝瑶寨被列为全国旅游扶贫示范项目，116个村列为全国乡村旅游扶贫重点村。2．87万名贫困对象纳入政策兜底。平安创建举措更实。强力开展严打整治飓风、亮剑行动和集中打击“盗抢骗”犯罪等专项行动，全面推行公安武警武装联勤巡逻、城区网格化巡防、常态化夜巡查缉和农村“一村一警”，积极发展一万名义警队伍，推动万人巡防，扎实抓好公安机关“一平台五中心”和省市县际治安站卡建设，切实加强道路交通、消防、治安等领域公共安全和企业生产安全监管，平安永州建设再上新台阶，综治工作再获全省先进，公安工作主要指标保持全省领先，全年没有发生一起较大以上安全事故，人民群众安全感、幸福感持续提升。

五年奋发进取，五年成就斐然。这一切，凝聚着上级党委、政府的亲切关怀，凝聚着市委团结带领全市人民的艰苦拼搏，凝聚着离退休老领导、老同志的热情关心，凝聚着社会各界的大力支持。在此，我代表市人民政府，向所有为永州改革发展付出辛劳智慧，对市人民政府工作给予支持帮助的同志们、朋友们，表示衷心的感谢，并致以崇高的敬意！

五年探索前行、砥砺奋进，给我们以宝贵的启迪和昭示。这就是：无论条件怎么艰难，保持“三线城市干一流业绩”的坚定信念，把更多的不可能变成可能，把更多的偶然变成必然，始终是我们工作的力量源泉；无论形势怎么变化，保持“一张蓝图干到底”的强大定力，一以贯之抓落实，不达目的不罢休，始终是我们工作的成功之道；无论任务怎么繁重，保持“一事当前、舍我其谁”的责任担当，一级带着一级干，一级做给一级看，始终是我们工作的有力保障；无论困难怎么叠加，保持“勇于创新、敢于争先”的昂扬锐气，不断以思维创新破解前进中的难题，以思想解放促进新起点上的发展，始终是我们工作的强劲动力。

成绩令人鼓舞，经验弥足珍贵，问题和困难更让我们警醒：经济总量不大、结构不优、动能不强的状况没有根本改变，企业生产经营整体不容乐观，经济下行压力持续加大，部分指标存在差距；民生和社会事业短板较多，群众普遍关心的上学难、就业难、看病难等问题仍未有效解决，脱贫攻坚任务繁重，社会不稳定因素仍然较多；政府职能转变、干部工作作风、依法行政能力与新常态、新形势的要求还不相适应，损害群众利益的腐败现象、行业不正之风和为官不为行为依然存在，发展环境还有待改善。对此，我们将高度重视，认真对待，着力解决，决不辜负全市人民的期望和重托。

二、未来五年工作的总体思路和主要目标

今后五年，是我市适应经济发展新常态的转型期、全面建成小康社会的决胜期。政府工作总的指导思想是：紧密团结在以习近平同志为核心的党中央周围，高举中国特色社会主义伟大旗帜，深入学习贯彻习近平总书记系列重要讲话精神，认真落实“四个全面”战略布局和“五大发展理念”，切实按照省第十一次党代会和市第五次党代会作出的决策部署，以全面建成小康社会为总抓手，以“力争在建设富饶美丽幸福新湖南的生动实践中走在前列”为总要求，大力实施创新引领、开放崛起战略，全力打好城镇提质、转型升级、脱贫攻坚、环境治理等战役，加速建设品质活力新永州、创新开放新高地。

主要奋斗目标是：

——提前建成全面小康社会。保持经济中高速增长，主要指标增速快于全省平均水平，城乡居民人均可支配收入增速快于经济增速。到2021年，全市地区生产总值达到2700亿元，财政总收入达到300亿元，社会消费品零售总额突破1000亿元，规模工业增加值突破600亿元，固定资产投资累计完成1万亿元以上。2019年全面建成小康社会。

——夺取脱贫攻坚全面胜利。全力推进精准扶贫、精准脱贫，到2019年，贫困地区农民人均可支配收入突破1万元，现行标准下建档立卡贫困人口全部脱贫、贫困村全部出列、贫困县全部摘帽。

——初步建成区域性综合交通枢纽城市。以构建区域性铁路枢纽城市为核心，统筹推进高速公路、干线公路、航空、水运建设，加快建成出省跨境大通畅、县乡公路大循环、旅游景区大连接的综合立体交通网络，基本实现市到县、县与县之间以高速公路或一级公路相连接，中心城区至南部六县形成一个半小时交通圈，南部六县之间形成一小时交通圈。区域性铁路枢纽城市建设，争取衡柳高铁提速，新建呼南高铁邵永段、永贺高铁、兴永郴赣铁路，实现多条铁路干线在永州融会贯通，推动永州铁路网从“十字架”到“米字型”的转变，构建与邻省主要城市3小时高铁经济圈；抓好湘桂铁路永州地区扩能改造工程，打造综合客运枢纽和铁路物流中心；加快编制中心城区轨道交通发展规划，推进湘南地区城际轨道交通可研工作，积极配合省里和广东，开展怀永清（广州）高铁前期规划研究。公路、航空、水运建设，突出抓好衡道高速、永郴高速、永（州）新（宁）高速、零陵机场迁建、江华护林机场、湘江千吨级航道改扩建、湘桂运河等重大工程前期工作或开工建设，强力促进一批国、省干线公路建设改造，扎实抓好县乡公路、农村公路提质升级。

——积极创建国家生态文明建设示范市。全面实施生态优先战略，认真落实最严格的水资源管理和节约用地制度，加强节能减排，扎实抓好湘江保护和治理“一号重点工程”，全面推行河长制，深入推进生态文明建设示范县区、示范乡镇创建和美丽乡村建设，加快完善生态产业结构和制度体系，推动形成绿色低碳生产生活方式，努力建成天更蓝、地更绿、水更清、城乡更美、宜居宜业、国内知名的生态文明城市。

——成功创建全国文明城市。坚持城市硬件建设与软实力提升两手抓，大幅提升中心城区的综合承载力、辐射带动力，显著提高市民综合素质和城市整体文明水平。到2020年，成功跻身全国文明城市行列。

——基本构建潇湘城市群。推动中心城区提质扩容迈上更高层次，初步形成“一江两岸、东扩西跨、南北联城、中部崛起”的城市框架。以交通连城、产业兴城、生态融城为重点，加快区域融合发展步伐，基本构建以冷水滩、零陵为主中心，以祁阳、东安、双牌为支撑，具有区域核心竞争力和影响力的潇湘城市群“半小时经济圈”，辐射带动以宁远、新田、蓝山为主的“二广高速经济带”和以道县、江华、江永为主的“生态经济圈”协同发展，打造市域城镇“一核一群一带一圈”格局。力争将潇湘城市群培育成连接长江开放经济带和珠三角经济带的重要区域性城市群、区域物流中心、全省对接东盟的先行实验区，让一个更加充满活力、更具品质神韵的崭新永州，矗立于五岭之北，崛起在潇湘之滨！

实现上述目标，要突出抓好五个方面的重点。

（一）坚持创新引领，加快动能转换。始终把改革创新摆在经济社会发展的核心位置，贯穿于各领域各环节。大力实施创新驱动发展战略，持续推进大众创业、万众创新，扎实抓好新型工业化、信息化、城镇化、农业现代化、绿色化“五化协同”，更好落实投资、消费、出口“三轮驱动”，积极对接“中国制造2025”和“互联网＋”行动计划，全面改造提升传统产业，加快培育壮大新兴产业，着力构筑多点支撑的产业发展新格局，推动经济发展由规模、要素驱动向创新、效益驱动加速转换。五年内，传统产业和新兴产业均建成千亿产业，永州经开区建成国家级开发区，长丰汽车、零陵烟厂建成百亿企业。现代农业、现代服务业和高新技术产业发展跃上新台阶。

（二）狠抓统筹协调，拉长发展短板。瞄准经济社会发展薄弱环节，加大统筹协调力度，着力在协调发展中拓宽发展空间，在加强薄弱领域中增强发展后劲。以做大做强中心城市和潇湘城市群为重点，加快形成“一核一群一带一圈”的发展格局。推进以人为核心的新型城镇化，探索建立城乡发展一体化机制。加快建设文化强市，着力构建现代公共文化服务体系和现代文化市场体系，促进物质文明与精神文明协调发展。

（三）擦亮绿色名片，建设美丽家园。坚持绿色富市、绿色惠民，大力开展环境污染综合治理，加大生态环境保护和自然生态系统修复力度，认真落实生态补偿机制，普遍推行垃圾分类制度，加快推进畜禽养殖废弃物处理和资源化，努力建设生态宜居、人与自然和谐发展的美丽永州。五年内，水、大气、土壤等环境质量明显改善，城市空气质量优良天数达到85％以上，森林覆盖率稳定在65％以上，湿地保护率稳定在70％以上，水功能区水质达标率保持100％。

（四）推进开放崛起，打造承接高地。充分发挥毗邻“两广”、对接东盟的区位优势，全面融入“一带一路”、“一带一部”大格局，积极推进双向开放和内外联动，加大招商引资和跨区域合作，加快对外贸易优化升级，深入实施走出去战略，全力建设好承接产业转移示范区，构建开放大平台和新机制，努力打造对接东盟桥头堡、“一带一部”重要战略支点和区域合作新典范。

（五）着力共建共享，提升民生福祉。牢固树立以人民为中心的发展思想，以持续增加城乡居民收入为核心，以建立更高水平的社保、住保、环保和就业、就学、就医“三保三就”为重点，着力解决人民群众最关心、最直接、最现实的利益问题，使发展成果更多更公平地惠及全体人民，让人民有更多获得感，努力建设社会安定有序、人民安居乐业的平安幸福永州。

各位代表，实现未来五年发展目标，重中之重是要认真落实省委要求永州在建设富饶美丽幸福新湖南的生动实践中走在前列的指示精神，紧紧围绕市第五次党代会描绘的宏伟蓝图，全面抓好与省第十一次党代会提出的目标愿景、战略举措、工作抓手的对接，全方位开展先行先试、创先争优，继续彰显“三线城市干一流业绩”的干劲和作为，努力为“新湖南”建设提供更多永州经验，增添更多永州能量，树立更多永州标杆。

三、2017年工作安排

2017年是实施“十三五”规划的重要一年，也是实现市第五次党代会宏伟蓝图的开局之年。我们要认真贯彻落实中央、省委、市委的决策部署，坚持稳中求进工作总基调，坚持以推进供给侧结构性改革为主线，全面做好稳增长、促改革、调结构、惠民生、防风险各项工作，掀起跨越赶超新热潮，迈开“走在前列”新步伐，以优异成绩迎接党的十九大胜利召开。

要努力实现：地区生产总值增长8．5％，规模工业增加值增长8．5％，固定资产投资增长14．5％，社会消费品零售总额增长12％，进出口总额增长12％，财政总收入增长12％，全体居民人均可支配收入增长8．7％，常住人口城镇化率提高2个百分点，全面小康实现程度提升3个百分点。全面完成环境保护、资源节约、安全生产等约束性指标。

（一）推进创新创业，增强发展动能

做大创新创业主体。完善鼓励创新创业政策支持体系，放宽民营经济投资领域和准入条件，积极支持社会资本通过PPP等方式进入公共服务领域投资创业。巩固省级创业型城市建设成果，用好“135”工程平台，深入开展创新创业带动就业示范县区和示范基地建设，大力促进高校毕业生就业创业，扶持农民工返乡创业。全年新设立市场主体2．5万个，新设立企业3000家，新培育小微企业1400家以上。

做强创新创业支撑。强化金融支撑。鼓励、引导金融机构加大对实体经济的支持力度，全年新增贷款185亿元，新引进1家股份制银行，设立4家村镇银行和4家以上全功能县域支行。推进平台公司市场化转型发展，新组建市产业建设投资公司、市铁路建设投资开发公司，争取市级融资公司新增划转资产100亿元以上、融资到位200亿元以上。争取2家以上企业首发上市或新三板挂牌。争取市融资担保公司注册资本达到5亿元以上。积极抓好“险资入永”。强化科技支撑。争取实施省以上科技计划项目30项以上，全社会研发经费增长25％以上，新认定高新技术企业10家以上，争创国家或省级研发平台2家以上。加强装备制造、食品加工、生物医药等优势主导产业关键技术攻关，深入推进质量强市和品牌兴市战略，引导企业发扬“工匠精神”，不断提高核心竞争力。强化人力资源支撑。深入实施高层次人才培育引进计划，加强本土企业家队伍培养，实施新生代农民工职业技能提升计划，培育更多创新创业能人。

做优创新创业环境。营造更加便捷的政务环境。大力推行“互联网＋政务服务”，加快建成全市统一的大数据共享交换平台，全面构建一窗受理、一站式办结、一网通办、线下线上融合的政务服务平台体系。营造更加规范的市场环境。深入开展反垄断行为专项整治，健全完善全市统一的公共资源交易平台，加快电子交易系统建设，构建透明、规范、高效的交易平台体系。营造更加诚信的社会环境。加强“诚信永州”建设，着力健全守信激励和失信惩戒机制，健全失信“黑名单”和社会信用统一代码制度，构建“一处失信、处处受限”的信用惩戒大格局。营造更加浓厚的兴商氛围。突出良性互动，坚持“官”“商”交往有道，构建“亲”“清”新型政商关系。

（二）推进产业升级，厚植发展优势

加快工业转型升级。聚焦传统产业抓提质。加大企业技改力度，全年投入工业技改资金330亿元。推进加工贸易创新发展，着力做大做强轻纺制鞋业，打造国际品牌鞋业研发制造基地。加快以锰产业为重点的冶炼行业转型升级。聚焦新兴产业抓集群。设立新兴产业发展基金，大力发展先进装备制造、电子信息、生物医药、新能源新材料、节能环保等新兴优势产业，推进产业链发展计划，加快形成集群优势。聚焦骨干企业抓引领。大力实施龙头企业培育工程、规模工业企业转型升级计划和中小企业专精特新发展专项行动，全年新增规模工业企业100家以上，新增主营业务收入过亿元企业10家以上，力争长丰猎豹整车产量达到7万台。聚焦园区平台抓集聚。加快实施百亿园区推进计划，年内引进100个重点项目落户园区。加快推进特色产业园区建设，市里集中力量打造信息产业园、新能源汽车及配套产业园，各县区因地制宜建好一个专业特色示范园区。聚焦永州智造抓转型。推进工业化、信息化深度融合，促进“永州制造”向“永州智造”跨越。制定“互联网＋制造业”融合发展路线图，积极开展互联网与工业融合创新试点，培育一批数字化车间和智能工厂。聚焦难题破解抓帮扶。实施重点产业、骨干企业精准帮扶行动，“一业一策”、“一企一策”解决用钱、用地、用能、用工等实际问题，在增强企业发展活力中促进工业转型升级。

加快现代农业发展。以增加绿色优质农产品供给为目标，深入推进农业供给侧结构性改革，打造以精细农业为特色的农副产品供应基地。狠抓标准化生产。优化农产品生产布局，加快建设一批现代规模农业基地和农业园区，着力将永州国家农科园、祁阳国家现代农业示范园打造成现代农业园区建设样板。抓好标准化果园、茶园、菜园、中医药特色药材园和养殖示范场等示范创建，新建农业标准化示范基地60个以上，新培育一批家庭农场示范场、农民专业合作示范社和供销基层标杆社。狠抓品牌创建。打响永州粮食品牌，确保总产稳定在315万吨左右。保持现代烟草农业领先地位，确保种植烤烟25万亩。做强做优香型农业、富硒农业和以国家地理标志保护产品为主的地方特色品种。新发展“三品一标”产品70个以上，新创省级著名商标20件以上。狠抓质量安全。推进农业生态绿色生产，实施化肥、农药使用量零增长行动，完善农产品防伪溯源体系和质量保障体系，创建国家出口食品农产品安全示范市，申报国家富硒产品重点实验室，建设平安农机示范市。狠抓电商助农。完善农村电商网络，大力开展“农产品上行”，推动“永品出永”更快发展。新创1＆shy；＆shy；—2家国家、省级电商示范基地或电子商务进农村示范县。拓展“互联网＋商贸流通”，打造“工业品下乡”和“农产品进城”双向流通渠道。搞好第三次全国农业普查。

加快服务业水平提升。全力推进全域旅游。启动以“锦绣潇湘”为品牌的全域旅游基地建设，打造国内知名旅游目的地。加快零陵古城建设，力争用三年时间打造成永州的会客厅、湖南旅游和文化的新标杆。积极拓展“水云潇湘·一江十景”和“四园一区”生态旅游文化品牌，打造中心城区“田园休闲区、城市后花园”。支持宁远、东安、江永等县区创建国家全域旅游示范区、双牌创建“国际慢城”，推进智慧旅游示范县区和示范景区创建。抓好乡村旅游富民工程，新培育10个乡村旅游示范村，开发一批乡村旅游精品线路。加快打通与周边城市旅游快速通道，积极融入湘粤桂旅游圈。加强等级景区建设，年内新创3家3A级景区，推进九嶷山创5A工作。加快发展文化体育健身产业。以创名城成功为契机，加强策划、宣传，打响永州国家历史文化名城品牌。深度做好文化与旅游、体育、饮食等的融合文章，充分释放名城品牌效应，全面提升永州文化的价值力、影响力和行销力。加快永州文化创意产业园建设，推动出版印刷、广告创意、广播影视等文化产业加速发展。启动永州水上运动训练基地建设，争取全国性体育训练基地或赛事落户。加快构建公共体育服务体系，发展健身休闲产业。加快发展现代物流业。围绕打造湘粤桂核心商贸流通枢纽，加快市县城市综合体、城市商圈、特色商业街区、大型专业市场建设，推进万商红商贸新城、永州内陆港、永州火车站物流园、潇湘汽车城等项目建设。引入第三方物流和互联网技术，提升快递仓储物流配送能力，支撑电商规模化发展。加快发展养老健康产业。坚持医养融合，鼓励公立医院和社会力量兴办养老机构、开展养老服务。支持永州职院、滨江新城健康产业园建设。新建一批森林康养示范基地。加强各类养老机构监管。拓展服务业发展领域。突出抓好金融、保险、会计、法律、中介、咨询等高端服务业发展。

（三）推进改革开放，激发发展活力

加速释放改革红利。着力在供给侧结构性改革上出实招。坚决落实去产能任务，稳妥化解冶炼、建材等行业过剩产能，有序处置“僵尸企业”。把房地产去库存与促进人口城镇化结合起来，着力增强中心城市和县城对农业转移人口的吸引力，继续用好鼓励农民进城购房等政策，加大高品质房源开发力度，释放和满足房地产市场新需求；做好市场开拓文章，抓好工业品去库存。高度重视去杠杆、防风险，积极争取政府存量债务置换政策，优化政府债务结构，加强金融机构监管，正确引导民间借贷。落实国家减税降费政策，大力降低企业融资、用工、用能、物流、各类交易特别是制度性交易成本，促进企业减负增效。加大补短板力度，聚焦城镇提质、产业发展、互联互通、社会民生等重点领域和薄弱环节，实施一批补短板工程。着力在“3＋2”专项改革上见成效。深化商事制度改革，全面推行集群注册、简易注销、营业执照全程电子化。推进行政许可标准化建设，最大限度精简行政审批事项，提升审批效率。全面完成农村承包土地经营权登记颁证工作，积极稳妥开展农村集体资产股份制改革试点和“三权分置”工作试点，推进农村承包土地经营权、林权抵押贷款，健全县乡农村产权交易平台。进一步创新网格化治理模式，推进城乡社区规范化、标准化建设，实施农村综合服务平台升级改造，大力培育社会组织，推进基层民主自治。深化财税金融和投融资体制改革，加快建立现代财政制度，加强财源培植，推进综合治税，深化拓展融资模式和融资方式。完善国有资产监管体制，确保国有资产保值增值。着力在原创性改革上求突破。针对经济社会发展薄弱环节，精心组织实施一批原创性改革试点，力争取得一批示范带动性强的改革经验和试点成果，贡献更多全面深化改革的永州样本。

全面提升开放水平。以更广的视野融入区域发展。全面深化对内对外开放合作，由重点承接珠三角、长三角产业转移向全面对接国家开放战略、省内区域发展和周边互利合作转变，加快构建全方位、宽领域、多层次的开放发展新格局。以更大的力度抓实招商引资。创新招商方式，突出小分队、点对点、产业链、专业化招商，以商招商、网络招商和商会招商。发挥贸促、侨联优势，扩大境外招商。实施招商项目“清单式”调度管理，加大签约项目落实力度，全年实际利用外资10．3亿美元，内联引资250亿元。以更高的标准做优对外贸易。大力实施优进优出工程，积极支持发展跨境电子商务，支持一批有实力的企业继续走出去，加速推进境外产业园区建设。持续优化通关平台，改善通关条件。全年实现外贸进出口总额10亿美元。

（四）推进“四网”完善，优化发展平台

织好交通网。继续抓好“三年交通大建设”，建成零双一级公路、东安芦洪市至小江口公路二期工程、祁阳黎家坪至冷水滩马坪等公路，加快推进冷东一级公路、宁远柏家坪至道县柑子园、道县上关至湘源温泉、江华分水岭至白芒营、沱江至涔天河等公路建设，抓紧实施永州铁路枢纽、湘江永州至衡阳千吨级航道改造、上大公路冷水滩农科园至植物园一级公路以及一批国道、省道和旅游公路建设改造工程。启动智慧交通建设。完成农村公路改造1163公里。抓好呼南高铁邵永段、永贺高铁、零陵机场迁建、衡道高速、永州综合交通枢纽等项目前期工作。

织好水利网。全面建成涔天河水库扩建工程，实现东安芦江水库下闸蓄水，加快推进涔天河灌区、毛俊水库、道县营乐源水库等在建水利工程建设和双牌水库等大中型灌区续建配套，抓好地质灾害防治、抗旱应急水源、湘江中心城区段生态治理及防洪工程，推进南津渡水库、新田金陵水库、道县向阳水闸等除险加固，全面完成四水治理、小农水、河流生态改造及五小水利等年度建设任务。实施农村饮水安全巩固提升工程，新解决155个贫困村饮水困难问题。抓紧零陵何仙观、宁远九嶷山、祁阳东方红等水库前期工作。

织好能源网。基本建成神华国华永州火电一期工程，做好二期工程前期工作。开工建设总装机59．4万千瓦的风力发电、总装机60兆瓦的光伏发电项目，实现总装机20万千瓦的涔天河电站投产发电。做好一批风电、光伏发电项目前期工作。开工建设2个110千伏输变电及风电、光伏、垃圾等发电电网接入工程，继续抓好农村电网升级改造。加快实施“气化永州”工程，力争永州至东安、双牌、道县天然气长输管线开工建设。

织好信息网。开工新建一批移动4G网络基站和小区宽带项目。实施“智慧永州”二期工程，加快华为云计算中心建设，引导云技术资源推广应用。推进国家电信普遍服务试点城市建设，积极争取国家“宽带乡村”示范工程和省农村宽带普遍服务项目试点。继续抓好户户通、村村响工程。

（五）推进城乡一体，强化发展支撑

强化龙头带动。突出以滨江新城、东部活力新城建设为重点，继续推进中心城区扩容提质，全年实施重点项目150个，完成投资100亿元以上。滨江新城着力抓好以零陵机场迁建，玫瑰湾片区、万商红片区开发，湘江东岸生态治理、永州大道后续配套、阳明大道建设工程为重点的“一场二片三线”和“一宫两馆六中心”建设，加快实现“中部崛起”。东部新城突出抓好二广高速永州东连接线、路网管网、品牌医院学校、水系治理、生态公园廊道、安置小区等基础设施建设，初步构建基本框架。加快现有城区提质步伐，着力抓好棚户区改造、主次干道绿化景观提质、风光带、地下综合管廊、重点人防工程等项目建设，进一步完善燃气、给排水、供电、信息、排污、停车场站等公共服务设施，加快推进凤凰园片供水、管网、人行道建设和改造，实现河东、河西同步发展。启动潇湘城市群建设，着力在零冷两区之间加快公共服务、基础设施建设，强化高端产业项目布局，促进两区相向发展，加快中心城区一体化；加快潇湘城市群联城快速通道建设，率先实现交通联城。加强和创新城市管理，巩固提升国家卫生城市、国家森林城市创建成果，实施“创交、创文、创园”攻坚行动，确保国家卫生城市复审合格、国家交通管理模范城市成功挂牌，力争创全国文明城市获得申报提名，创国家园林城市取得突破性进展。持续抓好宋家洲环境综合整治、小区整治，扎实做好全面禁止燃放烟花爆竹工作。加大违法建设防控、整治力度，坚决减存量、遏增量。坚持城市改造统一规划、统一实施，严禁滥挖乱改。加强建设工程质量监管，打造更多优质工程。

促进县域崛起。发挥承接产业转移示范区、对接东盟桥头堡平台优势，放大“蓝宁道新江”加工贸易走廊的集聚、辐射功能，加快县域开放发展。支持县区按照“因地制宜，优势优先”原则，培育壮大特色主导产业，加快形成特色产业集群。落实权限下放，强化用地、融资等要素保障，全力支持县区向上争取转移支付、申报项目资金、争创各类试点示范，支持在永金融机构网点向县域拓展，支持市县区融资平台开展合作。推进县城扩容提质，加快道路、管网等基础设施完善与配套，提高城市管理水平，创建更多国家、省级卫生县城、文明县城、园林县城。支持祁阳、道县加快撤县设市。推进扩权强镇，深化品质活力示范乡镇建设，新创一批国家、省级特色小镇、美丽宜居小镇、历史文化名镇和美丽乡镇示范镇。加强乡镇机构基础设施建设，改善基层工作、生活条件。强化村级经费保障，提高村、社区干部基本报酬。抓好村、居委同步换届。

打造美丽乡村。加快推进新农村建设和美丽乡村示范，新启动建设美丽乡村创建村60个以上，争取1—2个县区进入省新农村建设先进县区。深入推进农村环境综合整治，新上整治重点村476个。实施村容村貌“四清四化”工程，启动农村垃圾、污水处理整县推进工作，完成900个村的污水治理任务。加强农村规划编制，实现村庄规划全覆盖。

建设绿色家园。加强林地、湿地、自然保护区、水利风景区、野生动植物和古树名木保护管理，强化森林防火和林业有害生物防治，完成人工造林27万亩。力争将冷水滩四明山申报为国家森林公园，新创1—2个国家湿地公园。深入实施湘江保护和治理第二个“三年行动计划”，加快国家生态文明建设示范市、大气环境质量达标城市创建工作，开展第二次污染普查和土壤污染治理与修复试点。积极争取提高生态公益林补偿标准。抓好以国有林场为主的森林碳汇交易。完善国有林场改革。

（六）推进民生改善，共享发展成果

打赢脱贫攻坚战役。扎实抓好温氏养殖、光伏发电、电子商务、乡村旅游等产业扶贫项目，大力发展“一村一品、一户一业”等多元扶贫增收产业。完成扶贫贷款15亿元，推动贫困地区有条件的企业到资本市场直接融资。加快完善贫困村水、电、路、讯、房等基础设施和教育、科技、文化、医疗卫生等公共服务，全面落实扶贫兜底政策，抓实抓好劳务协作脱贫和“一户一就业”工作，完成易地扶贫搬迁4．4万人、贫困对象劳动技能培训1．3万人。加大驻村帮扶和党员干部结对帮扶力度，引导各类企业、社会组织、个人参与脱贫攻坚。全年实现减贫12万人以上，贫困村出列281个，宁远、双牌、江永脱贫摘帽。

完善社会保障体系。加快棚户区改造，全年完成城市棚户区改造4．74万户、农村危房改造4．26万户。坚持以创新创业带动就业，新增城镇就业5．7万人、农村劳动力转移就业4万人。实施全民参保登记计划，完善五项保险统一征缴制度，推进各项保险法定人员全覆盖。深化机关事业单位养老保险、收入分配等制度改革，继续提高企业退休人员、城乡居民养老金水平，提高城乡低保、特困人员供养标准和城乡医保财政补助，加快推进农村低保和扶贫标准“两线合一”，推动城乡居民医保异地就医即时结算。提升社会救助、社会福利、残疾人保障服务、养老服务、慈善事业等水平，精准有效救助孤儿、重残智残人员、流浪乞讨人员等困难群体，抓好农村敬老院、城乡养老示范点、农村留守儿童保护中心等福利设施建设。

加快社会事业发展。积极推进教育综合改革，持续抓好“三名工程”，加大乡村教师队伍建设力度，扩大免费定向师范生培养计划，加快普及高中教育。实施城区学位和公办幼儿园建设三年攻坚行动，今年新增城区中小学学位5万个，其中中心城区1．2万个，每个县区新建1所公办幼儿园，市里新建2—3所，2019年基本消除城区大班额问题和幼儿入学难题。继续改善农村办学条件，新建农村标准化学校60所、农村公办幼儿园20所。加快永州幼儿高等师范专科学校、永州职业技术学院本部二期、潇湘技师学院实训基地建设和永州工贸学校搬迁，依托永州电大构建永州开放大学，启动市委党校搬迁，支持湖南科技学院推进“双一流”建设。推进文化强市，加强文物保护和文化传承，实施文艺精品创作和演艺惠民工程，举办全市公祭舜帝大典和周敦颐诞辰千年纪念活动，支持道县申报省级历史文化名城。加快市档案馆扩建改造和永州数字档案馆建设，抓好《永州市志》编修和县区年鉴编纂工作。实施健康优先发展战略，加快完善公共卫生体系，加快市儿童医院、湘雅永州医院建设和市中心血站搬迁，开展基层医疗卫生机构标准化建设三年行动，提升中心城市医疗服务档次和县域综合医疗服务水平。深化医药卫生体制改革，加快构建“三医”联动机制和分级诊疗体系，推进名医（护）、名科、名院创建。加强重大疫病防控和疾病防治，创建“百岁健康市”。强化食品药品监管，大力开展学校、企业、单位食堂标准化建设年活动，扎实抓好市食品检验实验室和县级区域性食品检验检测中心建设，加快实施农贸市场和超市生鲜食品安全快检工程。坚持计划生育基本国策，完善与全面两孩政策配套的公共服务体系，加大人口出生缺陷综合防控。高度重视老龄事业，扎实做好老干部工作。认真落实民族、宗教、侨务政策。充分发挥工会、共青团、妇联等群团组织作用。加强国防动员和后备力量建设，推进军民融合深度发展。

提升社会治理水平。深入实施公民道德建设工程，加强高校和中小学思想政治工作，广泛开展良好家风培育。推进依法治市，扎实抓好“七五普法”，增强全民法治意识。深化社会治安综合治理，加强以网格化、信息化、法治化、实名制为重点的社会治安防控体系建设和虚拟社会管理。坚持严打方针不动摇，始终保持对刑事犯罪严打高压态势，对各类犯罪活动“零容忍”。持续开展整治“三强三堵”、“两抢一盗”、黑恶势力、黄赌毒等专项行动，依法打击和妥善处置非法集资、电信诈骗等涉众型犯罪，全面加强反暴恐工作和国家安全工作，强化重大事项社会稳定风险评估，全力维护政治稳定和社会安全。创新矛盾纠纷化解方式，完善“三调联动”体系。扎实推进信访“千案攻坚”，从严查处信访违法行为。深入开展打非治违，全面排查治理重大消防安全隐患和道路交通安全隐患，全力管控安全生产风险，坚决杜绝较大以上安全生产事故发生。

各位代表，站在新起点，承载新使命，实现新愿景，我们将更加注重自身建设，努力以严的标准、严的要求、严的措施，打造勤政务实、廉洁高效、人民满意政府。

坚定信念，忠诚履职。加强理论武装，深入学习贯彻习近平总书记系列重要讲话精神，坚决执行党的路线方针政策。始终保持对党绝对忠诚，牢固树立政治意识、大局意识、核心意识、看齐意识，坚决维护习近平总书记的领导核心地位，始终同以习近平同志为核心的党中央保持高度一致。认真落实省委、省政府和市委的决策部署，自觉在市委的统一领导下开展工作，确保政令畅通、令行禁止。

践行法治，依法履职。认真履行推进法治建设第一责任职责，带头严格遵守宪法、法律、法规，自觉运用法治思维和法治方式推动工作，把政府活动全部纳入法治轨道。坚持科学民主依法决策，严格落实重大决策程序制度，不断深化行政执法体制改革，深入推进立法工作。更加自觉接受人大法律监督、工作监督和政协民主监督，推动人大代表建议、议案和政协委员提案办理跃上新水平。加强与民主党派、工商联、无党派人士和各人民团体的联系，虚心听取意见、建议。不断完善政府内部层级监督和专门监督，强化行政监察和审计监督。全面推进政务公开，加强政府网站建设管理，打造“阳光权力”。

敢于担当，勤勉履职。强化政府绩效评估和行政问责，加强领导干部实绩卡管理和干部队伍执行力建设，深入治理为官不为、为官乱为和庸政懒政怠政行为，建立完善创先争优激励机制和改革探索容错纠错机制，旗帜鲜明为敢于担当的干部担当，为敢于负责的干部负责，充分激发各级干部干事创业热情，努力营造想干事、敢干事、能干成事的浓厚氛围。

正风肃纪，廉洁履职。扎实抓好全面从严治党形势下的从严治政，坚决贯彻执行中国共产党廉洁自律准则和纪律处分条例，严格遵守党风廉洁建设各项规定，始终做到干净用权、干净干事、干净做人。加大反腐倡廉力度，切实加强对权力集中、资金密集、资源富集部门和岗位的监管，继续开展“雁过拔毛”式腐败问题和“纠四风、治陋习”专项整治。坚决防止“文山会海”、滥考乱评等“四风”问题反弹，推动作风不断好转。

各位代表，新征程赋予新使命，新蓝图召唤新作为。让我们在市委的坚强领导下，不忘初心，继续前进，为如期建成全面小康社会、开创永州更加光明灿烂的前景而努力奋斗！
